# Topical application of jojoba (*Simmondsia chinensis* L.) wax enhances the synthesis of pro-collagen III and hyaluronic acid and reduces inflammation in the *ex-vivo* human skin organ culture model

**DOI:** 10.3389/fphar.2024.1333085

**Published:** 2024-01-26

**Authors:** Zipora Tietel, Sarit Melamed, Navit Ogen-Shtern, Noy Eretz-Kdosha, Eldad Silberstein, Tomer Ayzenberg, Arnon Dag, Guy Cohen

**Affiliations:** ^1^ Food Science, Gilat Research Center, Agricultural Research Organization–Volcani Institute, Rishon LeZion, Israel; ^2^ The Robert H. Smith Faculty of Agriculture, Food and Environment, The Hebrew University of Jerusalem, Jerusalem, Israel; ^3^ The Skin Research Institute, The Dead-Sea and Arava Science Center, Masada, Israel; ^4^ Ben Gurion University of the Negev, Eilat, Israel; ^5^ Department of Plastic Surgery, Soroka University Medical Center, Ben-Gurion University of the Negev, Beer-Sheva, Israel; ^6^ Fruit Tree Sciences, Gilat Research Center, Agricultural Research Organization–Volcani Institute, Rishon LeZion, Israel

**Keywords:** jojoba wax, cosmetic applications, *ex-vivo* human skin, anti-inflammatory, collagen, hyaluronic acid, lipopolysaccharide

## Abstract

Jojoba (*Simmondsia chinensis* L.) wax was previously reported to increase cutaneous wound healing, ameliorate acne and psoriasis manifestations, and reduce oxidative stress and inflammation. However, its potential cosmetic properties have not been fully investigated. Thus, the current study aimed to evaluate the anti-inflammatory activities of jojoba wax and its impact on the synthesis of extracellular components following topical application. The fatty acid and fatty alcohol profiles of two industrial and two lab-scale cold-press jojoba waxes were analyzed along with total tocopherol and phytosterol content. The dermo-cosmetic effect of all jojoba wax preparations was evaluated *ex-vivo*, using the human skin organ culture model, which emulates key features of intact tissue. The ability of jojoba wax to reduce secreted levels of key pro-inflammatory cytokines and the safety of the applications in the *ex-vivo* model were evaluated. In addition, the impact on the synthesis of pro-collagen and hyaluronic acid levels upon treatment was investigated. The results demonstrate that topically applied jojoba wax can reduce LPS-induced secretion of IL-6, IL-8, and TNFα by approx. 30% compared to untreated skin. This effect was enhanced when treatment was combined with low non-toxic levels of Triton X-100, and its efficacy was similar to the anti-inflammatory activity of dexamethasone used as a positive control. In addition, mRNA and protein levels of collagen III and synthesis of hyaluronic acid were markedly increased upon topical application of jojoba. Moreover, the enhanced content of extracellular matrix (ECM) components correlated with the enhanced expression of TGFβ1. Collectively, our results further demonstrate that jojoba can reduce local skin inflammation, and this effect may be increased by emulsifier which increases its bioavailability. In addition, the finding that topical application of jojoba wax enhances the synthesis of pro-collagen and hyaluronic acid and may be beneficial in the treatment of age-related manifestations.

## 1 Introduction

Jojoba (*Simmondsia chinensis*) is an evergreen shrub native to the Sonoran Desert. Jojoba is currently cultivated in the US, India, Chile, Peru, Argentina, Australia, and Egypt, as well as in Israel, which is one of the leading growers (Benzioni, 2006; Perry et al., 2021). Jojoba seeds contain 48%–53% liquid wax, comprised of fatty esters (Tietel et al., 2021a). The wax’s chemical composition includes C_16_-C_24_ fatty acids and fatty alcohols, resembling the human skin’s natural sebum, which consists of 2%–30% wax esters (Picardo et al., 2009; El Gendy et al., 2023). It also contains tocopherols, as well as high levels of phytosterols (Van Boven et al., 1997). Traditionally, jojoba wax (as crushed seeds) was used by native Americans of Baja California for skincare, e.g., for treatment of cuts, sores, bruises, and burns, including sun and windburn, or hair lubrication (Dary, 2008). In modern research, jojoba wax has been reported to possess anti-inflammatory and wound-healing bioactivities (Habashy et al., 2005; El Sherif et al., 2023). Additionally, it has also been shown that jojoba wax may form an efficient barrier that protects the skin surface, retaining moisture in the skin, as determined by transepidermal water loss evaluation (Stamatas et al., 2008). Jojoba wax does not easily penetrate the skin, and its activity is mainly manifested in the skin’s uppermost layers (Stamatas et al., 2008; Patzelt et al., 2012).

During the last decade, there has been an increased demand for evidence-based cosmetic and dermatological products with natural active ingredient, accompanied by empiric studies and pre-clinical evaluations. Due to the preference to restrict and reduce animal experiments, *in-vitro* bioassays, e.g., with cells, 3D-reconstructed skin or tissues, are favored. Yet, evaluating the bioactivity of jojoba wax in cell or tissue cultures is inherently challenging, and proper emulsion may be required to increase its solubility (Stamatas et al., 2008). In general, several techniques may facilitate the solubility of lipids, or lipids incorporated into formulas, to in the medium to enable the correct evaluation of bioactivity in a water-based environment. This includes using organic solvents (e.g., ethanol, methanol, or dimethyl sulfoxide (DMSO)), an addition of an emulsifier, using co-solvents (glycerin, PEG (polyethylene glycol)-400) and others.), or using surfactants (e.g., Tween 20 or Tween 80). However, in many cases, these might be insufficient, and at high concentrations, many are cytotoxic, negatively affecting the efficacy of the natural material which is under investigation (Dayeh et al., 2004; Johnson, 2004; Ravindran et al., 2011; Kaur and Mehta, 2017).

As most of the studies describing the dermo-cosmetic properties of jojoba rely on *in-vitro* data, which does not fully emulate the clinical outcome, the need to validate the results in other model systems is required. In addition, *in-vitro* data cannot be used to evaluate topical applications. When using human skin explants (an *ex-vivo*) for evaluation, both demands are met; studying topical treatments while using a relevant correlative model for a clinical effect, may be achieved (Gvirtz et al., 220). In this experimental system, the skin tissue is maintained in an air-liquid interface, as the dermal side is submerged in culture media while the epidermis surface is in contact with the air. Thus, allowing topical application of active ingredient or formulation (Ogen-Shtern et al., 2020). The purpose of the current study was to evaluate the anti-inflammatory activities of jojoba wax and the possible impact on the synthesis of extracellular components following topical applications. To achieve these aims, we opted to use the *ex-vivo* human skin model, establishing the evidence-based utilization of jojoba wax for skin conditions.

## 2 Materials and methods

Unless specifically specified, all chemicals were purchased from Sigma-Aldrich (St. Louis, MO, USA), and all tissue culture reagents were from Biological Industries (Beit Haemek, Israel). Jojoba waxes and respective seeds were generously provided by Jojoba Valley (Kibbutz Gal-On, Israel). Seeds were harvested on fall 2020 season, and wax was extracted shortly after harvested. Industrial cold-press jojoba wax was used (***JV12***, ***JV14***) as well as lab-scale expeller cold press was extracted in our lab (CA59, Komet, IBG, Moenchengladbach, Germany) from the same seeds (***JV13***, ***JV15***, respectively), so that from the same seeds both industrial and lab-scale waxes were available and evaluated. Wax samples JV12 through JV15 were deposited in ARO-Gilat for future reference, voucher number 00224.

### 2.1 Jojoba fatty acid and fatty alcohol profiling

Jojoba fatty acid and fatty alcohol profiling was performed based on our previously reported method (Tietel et al., 2021b)**.** Wax samples (50 µL) were added to a 2 mL Eppendorf tube. One hundred and 50 µL of 3.2% sodium methoxide in methanol (w/v) were added and vortexed. Tubes were shaken in a thermoshaker (700 rpm) at 40 °C for 30 min. Next, 100 µL of double-distilled water (DDW) was added, followed by an addition of 1,000 µL hexane. The tubes were centrifuged for 2 min at 17,000 G. iEght hundred µL of the supernatant were then transferred to an injection vial supplemented with 200 µL of C17:0 (1 mg/mL, internal standard). Analyses were performed in triplicates (n = 3), each one from 100 gr of different seeds.

### 2.2 Chromatographic conditions

The samples, at 1 μL, were injected into an Agilent Technologies gas chromatograph (model 7890N) equipped with a mass spectrometer detector (model 5977). The carrier gas was helium, at a flow rate of 1 mL/min, on a DB-23 (60 m, 0.25 µm, 0.25 mm) column. The oven temperature was initially 175 °C for 5 min, then increased to 240°C at 5°C/min, and held for 9.5 min. Inlet temperature was 250 °C and split ratio 10:1. Fatty acids methyl esters (FAMEs) were identified by comparing retention times with those of standard compounds (FAMEs mix, Supelco, Sigma-Aldrich, Rehovot, Israel). The relative composition of the fatty acids in the waxes was determined as a percentage of total fatty acids.

### 2.3 Emulsifier optimization, preparation, and evaluation

For this part, several common emulsifiers in cosmetic products were evaluated, including Triton X-100, Tween-20 and Tween-80. Emulsifiers were prepared by mixing in concentrations of 1%–5% (w/w) in jojoba wax, and vortexing for 1 min until completely dissolved. Upon optimization (see in the results section), Triton X-100 was chosen as the preferred emulsifier, in concentration of 3%. Thus, in a 2 mL Eppendorf tube 30 mg Triton X-100 was weighed, and jojoba wax was added according to the chosen proportions (Table 1). The tube was mixed vigorously (Vortexer, Heathrow Scientific, IL, US) for 5 min, at 3,000 Hz. Then, double distilled H_2_O (DDW) was added (in amount according to Table 1), and the mixture was shaken at 3,000 Hz for another 10 min at room temperature. All wax and emulsion samples were kept in room temperature for the duration of the experiment, temperature effect on stability was not evaluated due to the heat-sensitivity of the active ingredients. In the bio-evaluation, the jojoba cultivars were diluted 1:10 immediately before application to reach a final concentration of 0.3% Triton X-100. A scanning microscopy was used to evaluate wax distribution and size (Supplementary Figure S1). All images were captured with a Nikon Eclipse 80i microscope (Nikon, FL, USA) operated by NIS-element Documentation software, and pictures were taken using a color digital camera (DS-Ri2, Nikon).

**TABLE 1:** Tested proportions for jojoba wax formulation optimization.

	Preparation A	Preparation B	Preparation C	Preparation D
Emulsifier (mg)	30	30	30	30
Jojoba wax (mg)	200	400	600	800
H_2_O (DW) (mg)	770	570	370	170

### 2.4 Chemical evaluation

#### 2.4.1 Total tocopherol content

The wax samples (300 μL) were weighed into an Eppendorf tube, and 700 μL ethyl acetate were added, followed by 200 μL of FeCl_3_ solution (0.2% in ethanol (w/v)), and vortexed. Then, 200 μL of 2,2′-Dipyridinyl solution (0.2% in ethanol (w/v)) were added and mixed again. Tubes were then shaken in a thermoshaker (Thomas Scientific, Grant-bio, NJ, USA) at 25°C and 700 RPM for 20 min, covered with an aluminum foil. Two hundred μl were then added to a 96-well plate in three replicates, and samples read with a spectrophotometer (Thermo Fisher Scientific, MA, USA) at 520 nm (Tietel et al., 2019). A six-point calibration curve based on 0–0.1 mg/mL concentrations of alpha tocopherol was used for calculations.

#### 2.4.2 Total phytosterol content

The wax samples **(**200 μL) were weighed into an Eppendorf tube, and 400 μL ethyl acetate were added and vortexed. In each of three replicates, 100 μL sample were added, followed by 100 μL Liebermann–Burchard (LB) reagent at −20 °C (made with 10 mL acetic anhydride and 1 mL H_2_SO_4_). Plates were covered with a designated plate sticker, covered with aluminum, foil and incubated at room temperature for 90 min, and then read at 675 nm (Tietel et al., 2019). Asix-point calibration curve based on 0–2 mg/mL concentrations of beta-sitosterol was used for calculations.

### 2.5 Human skin organ culture

The skin tissues were obtained from healthy female donors, between the ages of 40 and 65, who underwent elective aesthetic abdominal surgeries, and signed an informed consent form. The experiments were conducted with the approval of the IRB (Helsinki Committee) of Soroka Medical Center, Beer Sheva, Israel (Number 0258-19-SOR). We confirm that all experiments on the skin tissues were approved by the IRB committee, in accordance to the Helsinki declaration, and after informed consent, and that all methods were performed in accordance with the relevant guidelines and regulations. Human skin culture preparation and treatments were performed under aseptic conditions. Following the surgery, the tissue was placed in Dulbecco’s Modified Eagle Medium (4,500 mg/L glucose) supplemented with 100 μg/mL penicillin and 100 μg/mL streptomycin and was transferred to the laboratory within 3 h at 2ºC-8°C. The tissue was than placed in a laminar hood, and the subcutaneous fat layer was removed by scalpel. A designated mechanical press apparatus was used to process the skin into 0.64-cm^2^ pieces with an average thickness of 2.2 ± 0.36 cm, as previously described (Cohen et al., 2023a; Cohen et al., 2023b). The epidermal side was than sterilized by Pharma-C 70% Isopropyl Alcohol Wipes, and the tissues were placed in 6-well plates (4 pieces/well; 500 µL serum-free DMEM supplemented with 100 μg/mL penicillin and 100 μg/mL streptomycin) and left for recovery overnight in a humidified CO_2_ incubator, at 37°C.

### 2.6 LPS-induced skin inflammation, epidermal viability and morphological evaluation

The possessed human skin samples were transferred to new 12-well plates (one piece/well; 250 µL) containing growth media with lipopolysaccharide (LPS, 5 μg/mL) in the absence or presence of topically jojoba preparations applied, as is, or integrated with the emulsifier. Dexamethasone (10 µM), supplemented in the media, was used as a positive control for a reduction of inflammation. After 48 h, the viability of the epidermal layer, separated by heat (56°C; 1 min), was measured by 3-(4,5-dimethylthiazol-2-yl)-2,5-diphenyltetrazolium bromide (MTT), as previously reported (Wineman et al., 2015). The spent media from all test groups were collected, centrifuged, aliquoted and stored at −80°C until use. In addition, a similar set of treated skin pieces was fixed by formaldehyde (4%) for 24 h at 2–8 °C. Then, the tissues were washed twice with PBS and transferred to 70% ethanol until used. Following dehydration, 10 µm paraffin sections were prepared, and slides were stained with hematoxylin-eosin (H&E) solution, as previously described (Ogen-Shtern et al., 2020).

Measurements of specific markers of inflammation, interleukin (IL)-6, IL-8 and TNFα, in the collected spent media were quantified by commercial Enzyme-Linked Immunosorbent Assay (ELISA) kits (Biolegend, San Diego, CA).

### 2.7 Measurement of ECM components

Skin samples (0.64 cm^2^) were treated topically with Jojoba wax, without or with the emulsifier. After 24 h, one set of skin pieces from all test groups was harvested, and RNA was extracted (RNeasy Kit, QIAGEN), according to the manufacturer’s instructions. After nanodrop quantification, reverse transcription to cDNA and qPCR were performed with GoTaq 1-Step RT-qPCR System (Promega Wi, USA), according to the manufacturers’ instruction. The following primers were used:

Human Collagen type III - Forward primer CAT​GCC​AGG​TCC​TAG​GGG​AA; Reverse primer GAG​ACC​GTT​AGC​TCC​TGG​TT; Human GAPDH - Forward primer GGC​AAA​TTC​CAT​GGC​ACC​G; Reverse primer TCG​CCC​CAC​TTG​ATT​TTG​GA.

Secretion of pro-collagen, transforming growth factor beta-3 (TGFβ3) and hyaluronic acid was measure in spent media collected from a second similar set of treated skin pieces following treatment for 48 h. The quantifications were performed by ELISA kits (R&D systems, MN, USA; Mybiosource California, USA, respectively).

### 2.8 Statistical analysis

Statistical analysis was performed by JMP 16, using analysis of variance (ANOVA) with t-test for post-hoc pairwise comparison, significant at *p* < 0.05. All data generated or analyzed during this study are included in this published article (and its Supplementary Matrial S1).

## 3 Results and discussion

Several studies have investigated the medicinal properties of jojoba wax, including its dermo-cosmetics activities (Tietel et al., 2021a). Most reported studies used *in-vitro* models (cell culture-based) to demonstrate beneficial effects, such as anti-inflammatory activity and wound healing properties. Yet, these models struggle to solve low bioavailability of the active compound in hydrophilic environments which may interfere with the translation to clinical use, even after *in-vivo* evaluation in rodents. Others and we have used the *ex-vivo* human skin organ culture as a robust, non-animal, screening model that emulates several key activities of the skin tissue, including as a drug screening platform, to investigate local inflammation in normal and injured tissues, taking into account the limitations of an intact model (Danso et al., 2015; Corzo-León et al., 2019; Eberlin et al., 2021). One of the model’s strengths is the air: liquid interface used, allowing a topical administration of the compound on the epidermal layer, as used clinically. Thus, the primary goal of the current study was to use a clinically relevant non-animal replacement model to evaluate jojoba wax anti-inflammatory capacity as well as its possible skin rejuvenation properties.

**FIGURE 1:**
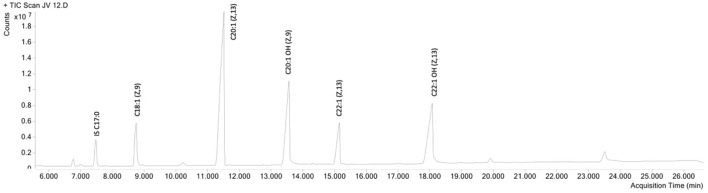
GC-MS chromatogram of jojoba wax, fatty acid methyl ester (FAME) method. IS-internal standard.

First, jojoba wax samples were obtained from two commercial batches (labeled JV12 and JV14), together with batch seed samples, from which their respective lab-scale batches (JV13 and JV15) were produced. Fatty acids and alcohols were profiled (Figure 1), and as can be seen in Table 2, the fatty acid and alcohol composition was generally similar between samples; Eicosenoic acid was the primary fatty acid, and fatty alcohols were equally distributed between C20:1OH and C22:1OH. These results align with other reported analyses of jojoba wax fatty acid and fatty alcohol profile (Awad et al., 2022). As for tocopherols, wax tocopherol levels varied between samples, with JV13 and JV15 showing the highest levels, followed by JV14, and JV12 showing the lowest levels (Table 2). At the same time, phytosterol levels were unchanged between samples. Jojoba tocopherol and phytosterol contents have scarcely been reported. Cold-press wax tocopherol contents was reported in a wide range of 63–417 mg/kg, while phytosterol contents was reported as 3.71–4 mg/gr wax (Van Boven et al., 1997; Tobares et al., 2003; El-Mallah and El-Shami, 2009; Shah et al., 2010). Tocopherols are potent lipophilic antioxidants, attributed, at least partially, to both the wax’s high oxidative stability and antioxidant activity (Caddeo et al., 2018). The results also suggest that for these parameters, laboratory and large-scale industrial preparations generally present similar phytochemical composition, which might imply that lab-scale samples generally resemble industrial material. Such similarity can be valuable in future evaluations of jojoba wax.

**TABLE 2:** Phytochemical composition of two commercial batches (labeled JV12 and JV14) and their two respective lab-scale batches (JV13 and JV15).

	JV12	JV13	JV14	JV15
Tocopherols (mg/kg wax)	91 ± 2 c	126 ± 2 a	101 ± 1 b	124 ± 1 a
Phytosterols (mg/kg wax)	1860 ± 20	1840 ± 30	1870 ± 20	1830 ± 30
C18:1 (%)	9.06 ± 0.27	9.15 ± 0.09	8.99 ± 0.18	9.08 ± 0.54
C20:1 (%)	70.4 ± 1.4	70.93 ± 0.9	70.29 ± 2.1	70.68 ± 1.1
C22:1 (%)	15.50 ± 0.25	14.73 ± 0.3	15.43 ± 0.21	15.06 ± 0.32
C20:1OH (%)	50.17 ± 0.9	49.84 ± 1.1	50.21 ± 1.6	52.97 ± 1.2
C22:1OH (%)	43.92 ± 0.91	43.24 ± 1.2	43.26 ± 1.2	40.66 ± 0.90

Presented values are mean ± standard error for three replicates (n = 3). Different letters denote a statistically significant difference between individual samples.

While sterols were not widely investigated as possible antioxidants, they have an important nutritional role in lowering blood cholesterol levels. These compounds are abundant in jojoba, which is why it was suggested as a cholesterol-lowering nutritional supplement (Tada et al., 2005). In addition, both these two groups of compounds have been shown to possess beneficial and protective effects on the skin, including anti-inflammatory bioactivities, thus imparting health-related properties, and considered quality parameters of jojoba wax (Miras-Moreno et al., 2016; Caddeo et al., 2018; Wang and Wu, 2019; Jiang et al., 2022).

**FIGURE 2:**
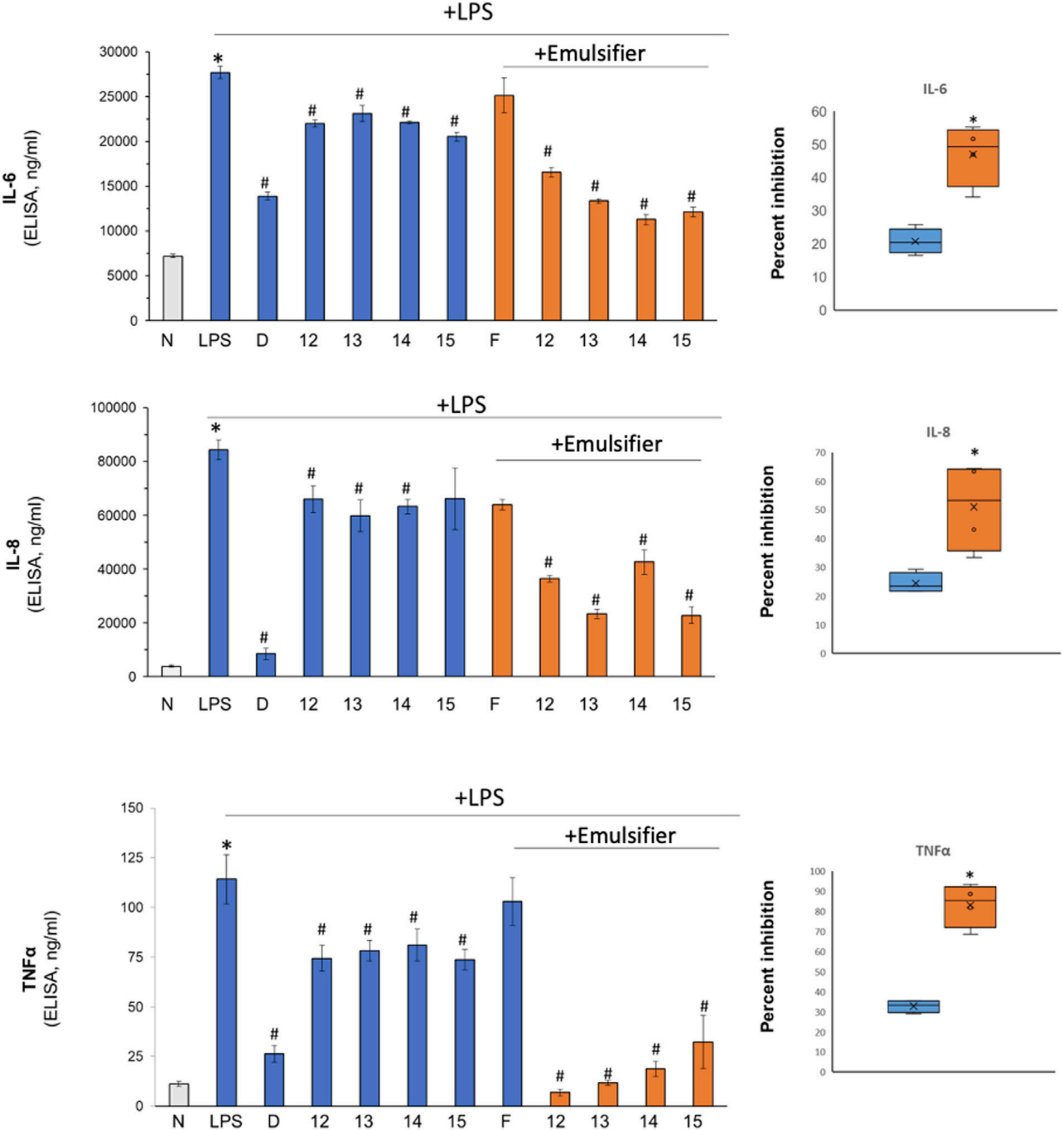
The impact of jojoba on LPS-induced skin inflammation. LPS (5 μg/mL) was added to the *ex vivo* human skin organ culture to induce inflammation. The tissues were treated without or with jojoba preparation (industrial cold-press jojoba wax (JV12, JV14) and laboratory preparation from the same seeds (JV13, JV15, respectively) at 850 μg/μL, in the absence or presence of the developed emulsion for 48 h. Then, the levels of IL-6, IL-8 and TNFα in the spent media were determined by ELISA. The right panel box depicts the aggregated comparison of natural vs emulsified jojoba wax. n = 3; *^/#^ *p* < 0.05 for significant difference from untreated and LPS-stimulated control, respectively. N- naïve tissue; F-formulated emulsifier vehicle control; D-dexamethasone (10 µM).

Next, jojoba waxes were evaluated for their anti-inflammatory action. Skin inflammation in the *ex-vivo* human skin organ culture was induced by LPS. As expected, a profound increase in IL-6, IL-8, and TNFα was seen upon LPS stimuli, which was reduced by the positive control steroid, dexamethasone (Figure 2). These cytokines were monitored due to their imperative activity in local skin inflammation and their high and repeatable response upon skin exposure to LPS (Argyropoulou et al., 2013; Kinn et al., 2015; Nosenko et al., 2019; Gvirtz et al., 2020). Importantly, topical application of jojoba wax at 850 μg/μL (pure jojoba) could reduce all three-cytokine hypersecretion by approx. 30%.

These results are aligned with two previous evaluations depicting an anti-inflammatory action of jojoba; Abdel-Mageed *et al.* (2014) have shown that jojoba leaf extract flavonoids can attenuate lipoxygenase activity, a key enzyme in arachidonic acid metabolism present upstream to synthesis of inflammatory mediators (Abdel-Mageed et al., 2014). In addition, a thorough *in-vivo* study by Habashy *et al.* (2005) showed that carrageenin-induced paw edema is also reduced by jojoba wax, which correlates with a significant reduction in prostaglandin E2 levels. In addition, the authors also showed a reduction of ∼30% in TNFα in the air pouch model (Habashy et al., 2005). Here, we elaborate and present a reduction of additional pro-inflammatory markers, IL-6 and IL-8, following stimulation with LPS. Still, the exact mechanism of action of the anti-inflammatory action and the reciprocal connection between prostaglandin and cytokine secretion upon jojoba application is unknown. Possibly, a feedback loop between a reduction in prostaglandin synthesis and cytokine secretion is observed here.

We hypothesized that the skin barrier properties reduce jojoba’s effectiveness, and by increasing the bioavailability of the wax or altering the dose of jojoba wax, more effective anti-inflammatory action will be presented. However, as jojoba wax could not be miscible in water or non-toxic levels of DMSO, a different approach was used; A laboratory-scale emulsifier preparation was generated. While both Tween-20 and Tween-80 jojoba preparations yielded unstable emulsions, Triton X-100 was found to give the most stable emulsion; all emulsifiers were evaluated at concentrations below their published toxicity levels (Dayeh et al., 2004; Ravindran et al., 2011; Patzelt et al., 2012; Kaur and Mehta, 2017). The stability was visually evaluated upon 5-min centrifugation at 17000G. Preparations that resulted in division into phases were eliminated. Formulation proportions were tested using a 3% emulsifier level, according to Table 1, using varying proportions of water and jojoba wax. The proportion of 80% wax: 17% H_2_O: 3% Triton X-100 (formulation D) was found to be the most stable (visual inspection). Stability was also confirmed by a scanning microscope, resulting in uniform small particle size (Supplementary Figure S1).

The efficacy of the jojoba preparation with Triton X-100 (emulsifier) was compared to natural jojoba wax effect on LPS-stimulated *ex-vivo* human skin. A preliminary study revealed that further 1:10 dilution in the emulsifier was adequate to increase the wax bioavailability concomitantly with the reduction of possible toxic side effects if used immediately. The results in Figure 2 (orange bars and right panel) clearly show that supplementation of 0.3% Triton X- 100 enhances the ability of jojoba wax to reduce LPS-induced secretion of pro-inflammatory cytokines; all three cytokines hypersecretion were further reduced by approx. 2-fold, presenting an efficiency equivalent to the positive control, dexamethasone. Interestingly, jojoba dry nano emulsion powders were recently shown to ameliorate LPS-induced acute lung injury and to reduce IL-6, IL-1β, and TNF-α levels (Zhang et al., 2021).

**FIGURE 3:**
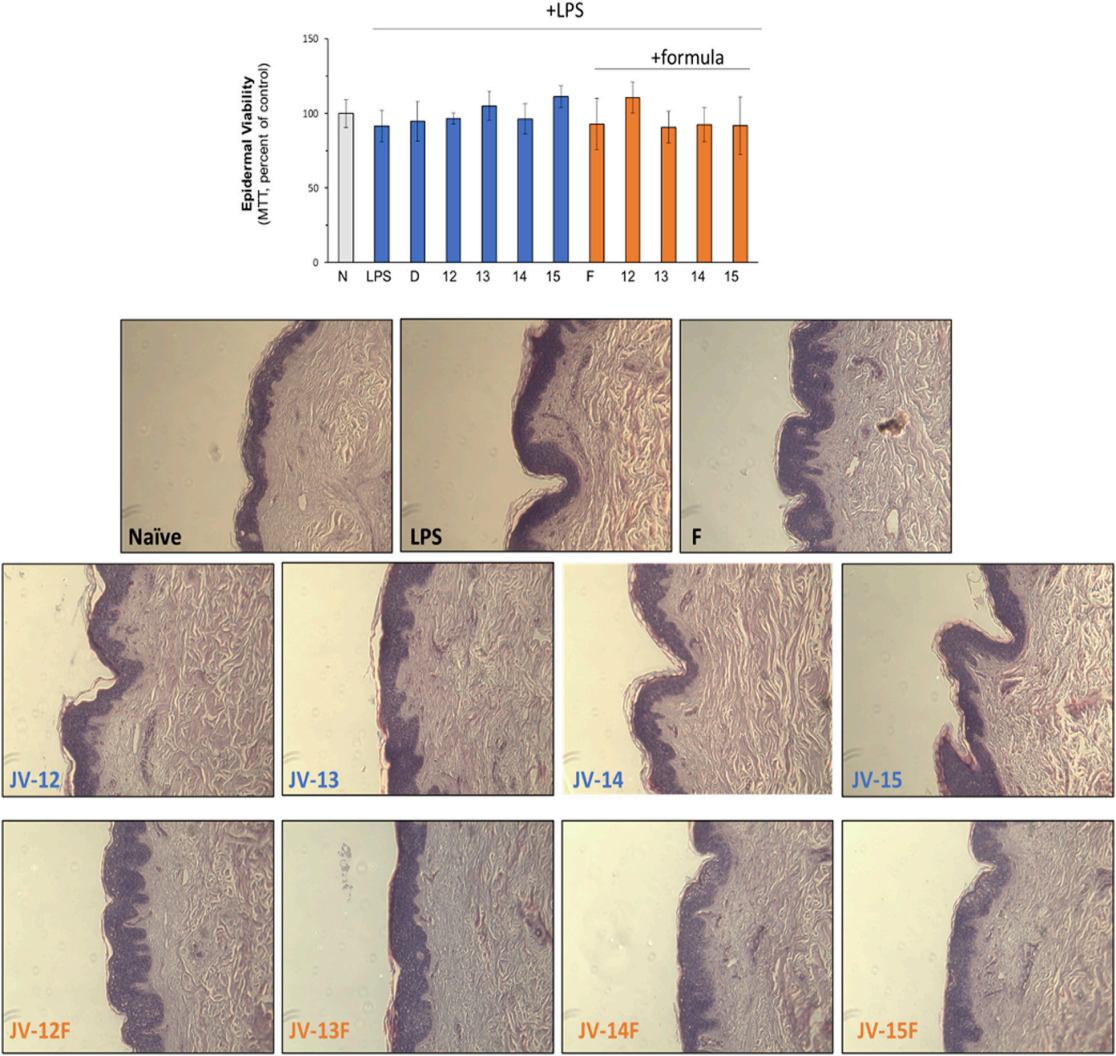
Safely evaluation of jojoba preparations. The skin tissues were treated as described in the legend of Figure 2. Epidermal viability was monitored by MTT (upper chart) and by histological evaluation. N- naïve tissue; D-dexamethasone (10 µM); F- formulated (emulsified) jojoba.

To exclude that natural or emulsified jojoba wax were toxic to the skin, several safety evaluations were performed. As can be seen in Figure 3, epidermal viability, as tested by MTT, was unaffected by neither jojoba nor its emulsified preparations. In addition, no morphological alteration was detected by histological examination, confirming that all treatment groups were non-toxic. These results support the findings of reduced levels of pro-inflammatory cytokines shown above, suggesting that the tissue was unharmed in all test groups.

The possible use of jojoba wax with low emulsifier levels has been indicated in previous works but was never demonstrated so far (Shevachman et al., 2004). The proposed method might have significant importance for future research on beneficial properties of jojoba wax performed in cell or tissue cultures for two main reasons: first, it improves the bioactivity of jojoba wax. Second, it is easy to implement and does not require special equipment. Therefore, using this method is an advantage for preparation in the laboratory scale.

**FIGURE 4:**
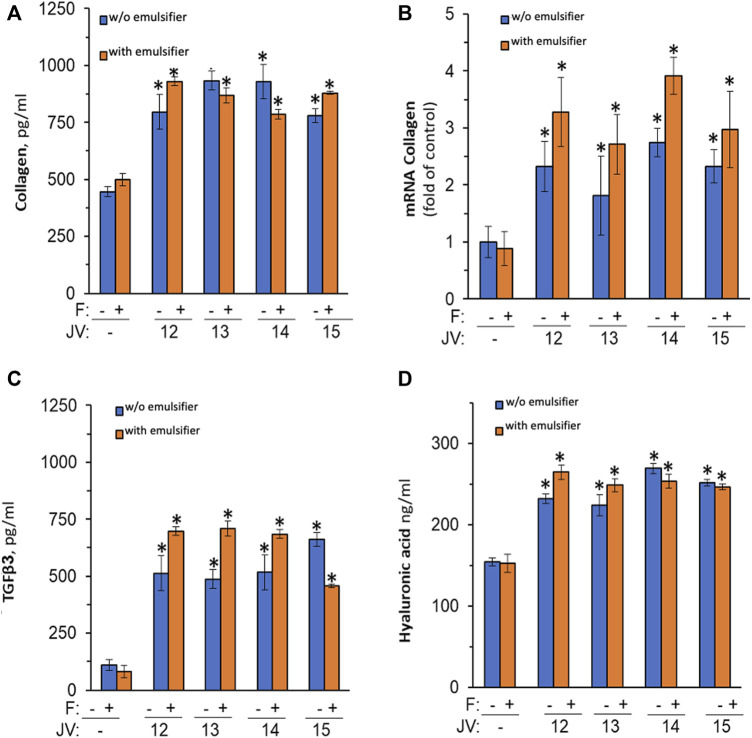
The impact of jojoba preparation on collagen and hyaluronic acid synthesis. The *ex-vivo* human skin organ cultures were treated without or with jojoba (JV) waxes (industrial cold-press jojoba wax (JV12, JV14) and laboratory preparation from the same seeds (JV13, JV15, respectively) at 850 μg/μL, in the absence or presence of the developed emulsion. Collagen **(A)**, TGFβ3 **(C)**, and hyaluronic acid **(D)** were evaluated in the spent media by ELISA. RT-PCR was used to assess collagen mRNA **(B)** after normalization to GAPDH levels. F-formulated emulsifier.

As lipids become an essential and innovative ingredient in cosmetics, with ever-increasing interest (Bonnet, 2018), lipid substance solubility is a significant issue. Although some authors have previously addressed it, available approaches are limited (Olivier et al., 2018). Attempts to incorporate jojoba wax in formulation required the use of a homogenizer, in addition to an overnight equilibration time (Shahin et al., 2011) or a high temperature of 65°C (Costa et al., 2014). Exposure of jojoba wax to such high temperatures was previously shown to result in deterioration of some of the active ingredients (Tobares et al., 2003).

few studies investigated the ECM remodeling properties of jojoba wax. For instance, Ranzato et al. (2011) have reported that jojoba can increase pro-collagen synthesis in fibroblast (Ranzato et al., 2011). Therefore, the *ex-vivo* system was used again, but without any exogenous stimuli. Topical application of all four jojoba preparations dramatically increases pro-collagen III synthesis (Figure 4A), which was not further enhanced by the emulsifier. In addition, a 2-fold increase in collagen mRNA was found by RT-PCR, linking the augmented levels of pro-collagen seen to the enhancement in gene regulation (Figure 4B).

One of the main regulatory factors of collagen synthesis is TFGβ (Carino et al., 2018). Therefore, the hypothesis that the jojoba-mediated pro-collagen enhancement was due to increased TGF secretion was investigated. Indeed, TGF-β3 levels were highly increased by all preparations of jojoba (Figure 4C), whereas the levels of β1 were below detection levels (data not shown). In addition, an augmented level of hyaluronic acid was observed in the same experimental conditions (Figure 4D). Thus, an additional ECM component that is modulated by the TGF signal pathway is therefore induced by jojoba. Unlike the increased efficiency that was observed for the anti-inflammatory effect, the emulsified wax had no significant added value in ECM synthesis, excluding an increased tendency to enhance collagen mRNA levels.

## 4 Conclusion

The current study demonstrates a marked anti-inflammatory effect and a significant enhancement in the synthesis of ECM components following treatment with natural and emulsified jojoba wax. In addition, a readily available method for a small-scale, low-cost preparation of a stable emulsion in the lab has been developed.

The dermo-cosmetic activities demonstrated by our findings join several other cosmetic parameters already shown to be improved by jojoba by other researchers (Blaak and Staib, 2022). These include, for instance, improved skin moisturizing and reduced trans-epidermal water loss (Stamatas et al., 2008), increased wound healing (Ranzato et al., 2011), anti-oxidant activity (Manoharan et al., 2016), and possibly battling skin pigmentation (Thawabteh et al., 2023), in addition to skin whitening (Arquette, 2006), and reduced comedones (Meier et al., 2012). These collective findings may shift the paradigm of jojoba usage from a structural ingredient in cosmetic formulation to a key active ingredient. The main novelty of the current study relays on several findings. First, supplementing jojoba wax with an emulsifier doubles its anti-inflammatory action. This may be of great interest for future studies on the anti-inflammatory action of the wax and its potential commercial applications. In addition, the ECM remodeling action, along with the observed increase in collagen III and hyaluronic acid synthesis has not been published and is in line with previous research, suggesting an increase in collagen I in jojoba-treated wounded fibroblast. Lastly, the demonstration of the effect on human skin, with a high correlation to clinical observations, strengthens the existing knowledge on the medicinal properties of the wax.

The use of the human skin organ culture in this study has several strengths stated above yet has its own limitations. For instance, the lack of circulating blood supply, the lack of systemic immune response or intact nervous system. Thus, further investigation that includes clinical studies is required in order to determine the nature of the active compound(s) as well as the full activity potential.

## Data Availability

The original contributions presented in the study are included in the article/Supplementary Material, further inquiries can be directed to the corresponding authors.

## Abbreviations

DDW, Double-distilled water; DMSO, Dimethyl sulfoxide; ECM, Extracellular matrix; ELISA, Enzyme-linked immunosorbent assay; FAMEs, Fatty acids methyl esters; H&E, Hematoxylin-eosin; IL-6, Interleukin-6; IL-8, Interleukin-8; LB, Liebermann–Burchard; LPS, Lipopolysaccharide; MTT, - 3-(4,5-dimethylthiazol-2-yl)-2,5-diphenyltetrazolium bromide, PEG 400- Polyethylene glycol 400; TGFβ, Transforming growth factor beta; TNFα, Tumor necrosis factor alpha.
