# Supplementary figures and images for: Topical application of jojoba (Simmondsia chinensis L.) wax enhances the synthesis of pro-collagen III and hyaluronic acid and reduces inflammation in the ex-vivo human skin organ culture model
Source: Front Pharmacol. 2024 Jan 26;15:1333085. doi: 10.3389/fphar.2024.1333085 (PMC10855461; doi:10.3389/fphar.2024.1333085)

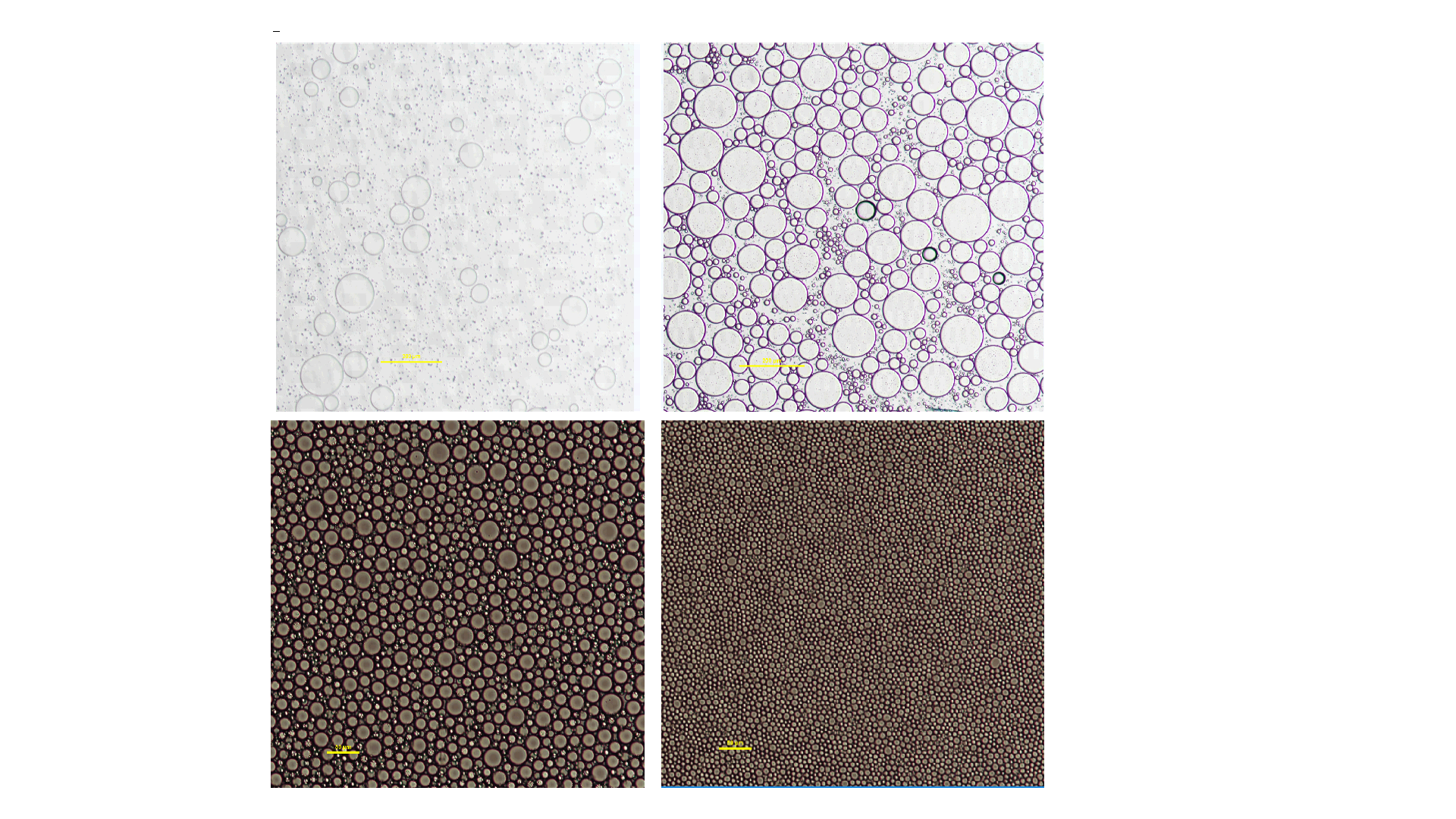

Supplement: Supplementary file 1 [file Image1.TIFF]
